# Proteomic Characterization of the World Trade Center dust-activated mdig and c-myc signaling circuit linked to multiple myeloma

**DOI:** 10.1038/srep36305

**Published:** 2016-11-11

**Authors:** Kai Wu, Lingzhi Li, Chitra Thakur, Yongju Lu, Xiangmin Zhang, Zhengping Yi, Fei Chen

**Affiliations:** 1 Department of Pharmaceutical Sciences, Eugene Applebaum College of Pharmacy and Health Sciences, Wayne State University, 259 Mack Avenue, Detroit, MI 48201, USA

## Abstract

Several epidemiological studies suggested an increased incidence rate of multiple myeloma (MM) among first responders and other individuals who exposed to World Trade Center (WTC) dust. In this report, we provided evidence showing that WTC dust is potent in inducing mdig protein and/or mRNA in bronchial epithelial cells, B cells and MM cell lines. An increased mdig expression in MM bone marrow was observed, which is associated with the disease progression and prognosis of the MM patients. Through integrative genomics and proteomics approaches, we further demonstrated that mdig directly interacts with c-myc and JAK1 in MM cell lines, which contributes to hyperactivation of the IL-6-JAK-STAT3 signaling important for the pathogenesis of MM. Genetic silencing of mdig reduced activity of the major downstream effectors in the IL-6-JAK-STAT3 pathway. Taken together, these data suggest that WTC dust may be one of the key etiological factors for those who had been exposed for the development of MM by activating mdig and c-myc signaling circuit linked to the IL-6-JAK-STAT3 pathway essential for the tumorigenesis of the malignant plasma cells.

Multiple myeloma (MM) is a malignant neoplasm of plasma cells localized within the bone marrow (BM) compartment and ranked second in prevalence of all hematopoietic malignancies^1^. In 2014, there were around 24,000 and 110,000 new cases in U.S and worldwide, respectively. Despite extensive effort was made in the past decades, the risk factors for MM are still elusive. Several earlier studies suggested that environmental exposures to industrial or agricultural products, such as benzene, petroleum products, and pesticides, may contribute to the development of MM^2^,^3^. Some recent cohort studies on the first responders, reconstruction workers and volunteers of the World Trade Center (WTC) after terrorist attack on September 11, 2001, provided evidence linking inhalation of the WTC dust to MM and other types of malignancy^4^,^5^,^6^. However, there are no studies revealing whether WTC dust is potentially carcinogenic, and if so, how WTC dust causes malignant transformation of the mature plasma B cells.

In the past decade, large-scale genomics studies have determined genetic landscape of MM and identified abnormal genetic events present in various disease stages, from premalignant monoclonal gammopathy of undetermined significance (MGUS) to smoldering multiple myeloma (SMM), active MM and relapsed MM^7^. The unique BM milieu is vital for the longevity of myeloma cells by providing various supportive BM cells and soluble factors^1^. Among these external stimuli, one of the most important factors is the interleukin-6 (IL-6). After binding to its receptor (IL-6R) and recruiting signal transducer, GP130 (also known as CD130 or IL-6ST), IL-6 can activate Janus Kinase (JAK)/signal transducer and activator of transcription 3 (STAT3), Akt and mitogen-activated protein kinase (MAPK) pathways to promote proliferation, survival and drug resistance of the MM cells^8^. Another hallmark of MM pathogenesis is the mutation- or overexpression-induced c-myc activation^9^. C-myc is a well-defined onco-protein involved in many types of human cancers. As an essential transcription factor, c-myc upregulates transcription of genes responsible for cell growth, proliferation and maintenance of cancer cell stemness^10^. In MM, c-myc overexpression can distinguish active MM from premalignant MGUS. In addition, activated c-myc has been shown to sustain the survival of myeloma cells^11^. More interestingly, a recent study indicates that collaboration between IL-6 pathway and c-myc results in significant acceleration of MM pathogenesis^12^. However, the underlying mechanisms of this oncogenic interaction remain unclear.

As a c-myc-induced protein, mdig (mineral dust-induced gene, also known as mina53, MINA, or NO52) functions as a histidyl hydroxylase and potentially a lysine-specific demethylase, which regulates gene transcription through modifying tri-methylated lysine 9 residue on histone 3 (H3K9me3)^13^,^14^. Consistent with this function, mdig is found to be exclusively localized in nucleus of various cell types^15^,^16^. Some studies have demonstrated that mdig exerts strong immune-regulatory function on T cells by favoring Th1 and Th17 cell differentiation in Th1/Th2, Th17/Treg balances, respectively^17^,^18^,^19^,^20^. Overexpression of mdig has been observed in many types of human cancer, including lung cancer, colon cancer, gastric carcinoma, etc.^21^,^22^,^23^,^24^,^25^,^26^. Meanwhile, mdig has been shown to be able to promote cancer cell proliferation^16^,^21^,^27^. Furthermore, mdig overexpression has been exclusively observed in various B cell-derived malignancies among major human lymphoma subtypes^28^, suggesting that mdig may contribute to c-myc-induced tumorigenesis in MM.

The WTC dust from the collapse of World Trade Center building due to terrorist attack is a mixture of toxic materials, many of which are know human carcinogens, including crystalline silica, asbestos, carbon nanotubes, lead, cadmium, mercury, dioxins, polycyclic aromatic hydrocarbons, etc^29^,^30^. By studying cancer incidence rate among workers of rescue, recovery, reconstruction, and volunteers who were exposed to the heavy dust cloud at Ground Zero and registered in the New York/New Jersey consortium of the WTC Medical Monitoring and Treatment Program (MMTP), Moline *et al*. noted 8 cases of MM, four of them were younger than 45 years^4^. This observation was supported by another longitudinal study of 55,778 New York State residents, which revealed excess incidence in responders for MM^5^. In the present report, we provide evidence revealing that WTC dust is potent in inducing mdig in normal B cells and MM cells and further demonstrating that mdig is significantly associated with the malignant transformation of MGUS to active MM, disease exacerbation and poor clinical outcomes. Biochemical studies unraveled that mdig directly interacts with c-myc and JAK1 in MM cells, which attributes to the hyperactivation of the JAK1 and STAT3 signaling important for cell survival, proliferation and development of drug resistance of the MM cells^31^,^32^. Taken together, our data suggest that mdig may serve as a key mediator for MM associated with WTC dust exposure and potential diagnosis/prognosis marker of MM.

## Results

### WTC dust induces mdig in bronchial epithelial cells, B cells and MM cells

**Figure 1:**
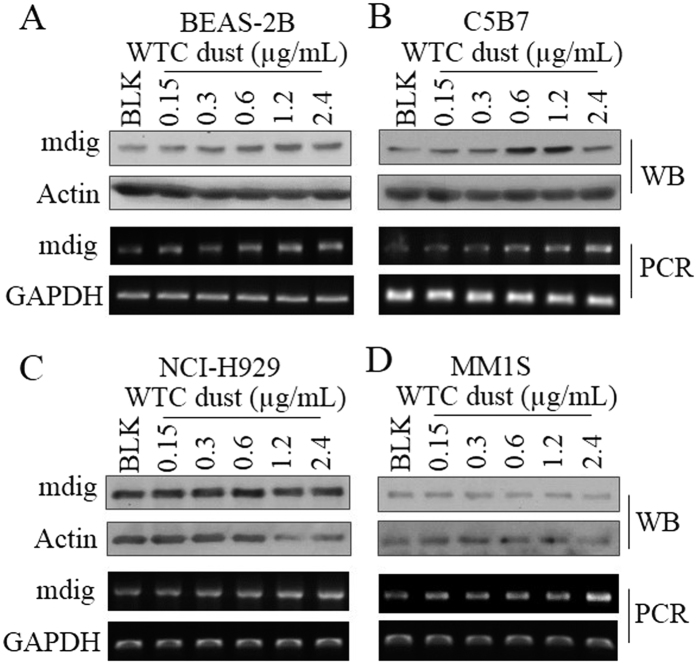
WTC dust induces mdig in BEAS-2B cells (**A**), C5B7 cells (normal **B** cells, **B**), NCI-H929 cells (MM cell line, **C**), and MM1S cells (MM cell line, **D**). All of the cells were treated with the indicated concentrations of WTC dust for 6 h, followed by Western blotting (top two panels) and RT-PCR (bottom two panels). Each panel is representative for at least three independent experiments.

The adverse effect of WTC dust on the respiratory system, including airway inflammation, impairment of the pulmonary function, airway hyperactivity, asthma, and sarcoid-like granulomatous pulmonary disease, had been well-established^33^,^34^. Indeed, we noted that WTC dust is highly capable of inducing mdig expression in the bronchial epithelial cell line, BEAS-2B cells, in concentrations ranged from 0.15 to 2.4 μg/ml (Fig. 1A). Since concerns had been arisen about the potential for increased risk of MM among WTC responders^4^,^5^, we also investigated the capability of WTC dust on the induction of mdig in normal B cells using a B cell line C5B7. Similar to what we observed in BEAS-2B cells, we noted a dose-dependent induction of mdig protein and mRNA by WTC dust in C5B7 cells (Fig. 1B). In two MM cell lines NCI-H929 and MM1S, although we did not detect significant induction of mdig protein, a pronounced induction of mdig mRNA by WTC dust was observed (Fig. 1C,D). These data, thus, clearly suggest that in addition to damaging the respiratory system through direct interaction, WTC dust or its components may influence the function or growth status of the B cells and the MM cells.

### Increased mdig expression in the bone marrow (BM) of the MM patient

**Figure 2:**
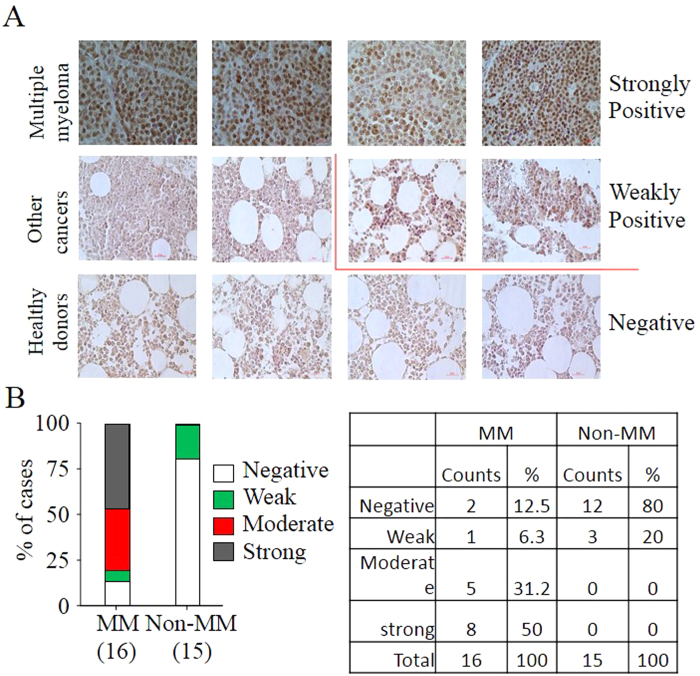
Increased mdig expression in human MM samples. (**A**) Representative IHC images of mdig expression in bone marrow (BM) of MM patients (n = 16), BM of non-hematological cancer patients (n = 11), and BM of healthy donors (n = 4). Magnification: 40×, scale bar: 50 μm. Strongly positive: over 50%; moderately positive: between 50% and 25%; weakly positive: between 25% and 5%; negative: less than 5%. (**B**) Summary of (**A**).

To determine whether mdig expression is clinically relevant for MM, we evaluated mdig protein levels in the BM specimens of MM patients through immunohistochemistry (IHC). In total of 16 cases of MM BM biopsies examined, 8 samples exhibited strong staining of mdig proteins as judged by the fact that more than 50% of cells are mdig positive, 6 samples showed moderate or weak mdig staining and 2 samples are mdig negative (Fig. 2A,B). We also checked another set of BM specimens collected from 4 healthy donors and 11 patients with other non-hematological cancers. Mdig protein was not detected in the BM specimens of all 4 healthy donors and 8 out of 11 cases of non-hematological cancer patients. Three BM specimens from patients with non-hematological cancers showed weak positive of mdig staining (Fig. 2A,B). Thus, these data indicate that the level of mdig may be associated with the onset of MM.

### Both mdig and c-myc are associated with disease aggressiveness of MM patients

**Figure 3:**
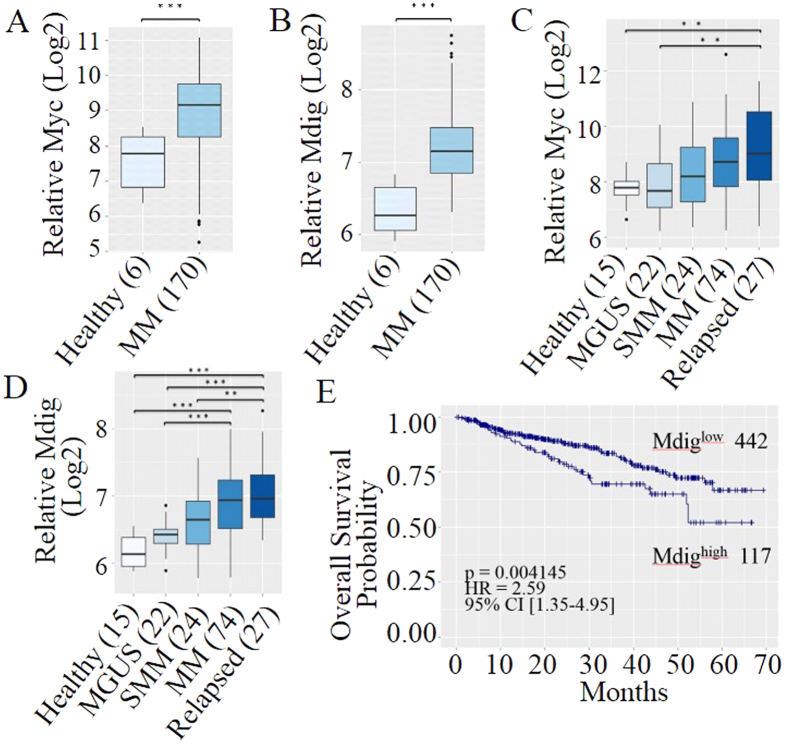
Overexpression of mdig and c-myc is associated with disease progression and poor prognosis of MM. (**A**) Box plot of relative level of c-myc mRNA in newly diagnosed MM patients and healthy donors (GES39754, n = 176); (**B**) Box-plot of relative level of mdig in newly diagnosed MM patients and healthy donors (GES39754, n = 176); (**C**) Expression level of c-myc mRNA in CD138^+^ plasma cells from healthy donors and MM patients at various stages (GSE6477, n = 163); (**D**) Expression level of mdig mRNA in CD138^+^ plasma cells from healthy donors and MM patients at various stages (GSE6477, n = 163). In the plots, boxes denote the inter-quartile range (25% to 75%), bars represent medians and whiskers indicate up to 1.5 × the inter-quartile range that covers 95% of all samples. Outliers are indicated by the black dots. Sample sizes of each group are annotated in parentheses and expression levels are displayed in log 2 scale. (***p < 0.001, **p < 0.01). (E) Kaplan-Meier (KM) survival curve of 559 MM patients (GSE2658) stratified by their mdig expression levels. Sample sizes of each group, log-rank p-value, hazard ratio and 95% confidence intervals are displayed in the figure. Tick marks on each arm represent censored samples.

There are several stages during disease development, including premalignant MGUS, asymptomatic smoldering MM (SMM), symptomatic MM, and relapsed MM^35^. It has been well-accepted that c-myc activation is a hallmark of MM pathogenesis^11^,^36^,^37^, especially in the early malignant transformation from MGUS to active MM^38^. C-myc has also been implicated in the up-regulation of mdig^27^. Overexpression of mdig has been observed in many types of human malignancies^13^,^14^,^21^,^22^,^23^,^24^,^25^,^26^,^27^,^28^, but its potential role in c-myc-related MM pathogenesis remains unknown. To determine whether mdig contributes to c-myc-induced MM pathogenesis, we first examined expression levels of mdig and c-myc in MM patients. We noted that both c-myc and mdig mRNAs are significantly up-regulated in newly diagnosed MM patients when compared to healthy donors (Fig. 3A,B). Further analysis of patients at continuous stages during MM development has demonstrated a robust elevation trend of both c-myc and mdig (Fig. 3C,D). Statistically significant increases of mdig mRNA, from MGUS to active MM and from SMM to relapsed MM were noted (Fig. 3D), suggesting a positive correlation between mdig expression and malignant transformation, disease progression and relapse of MM.

The involvement of mdig in MM pathogenesis is further supported by survival analysis of 559 MM patients^39^. High level of mdig expression is significantly correlated with poor overall survival of the MM patients, even though higher percentage of patients from mdig^High^ group (82%, 96/117) received intensive therapies than those from mdig^Low^ group (57.7%, 255/442) (Fig. 3E). Taken together, all above data demonstrate a strongly positive correlation of mdig and c-myc to the pathogenesis and aggressiveness of MM.

### Mdig acts as a key interaction partner of c-myc in MM cells

**Figure 4:**
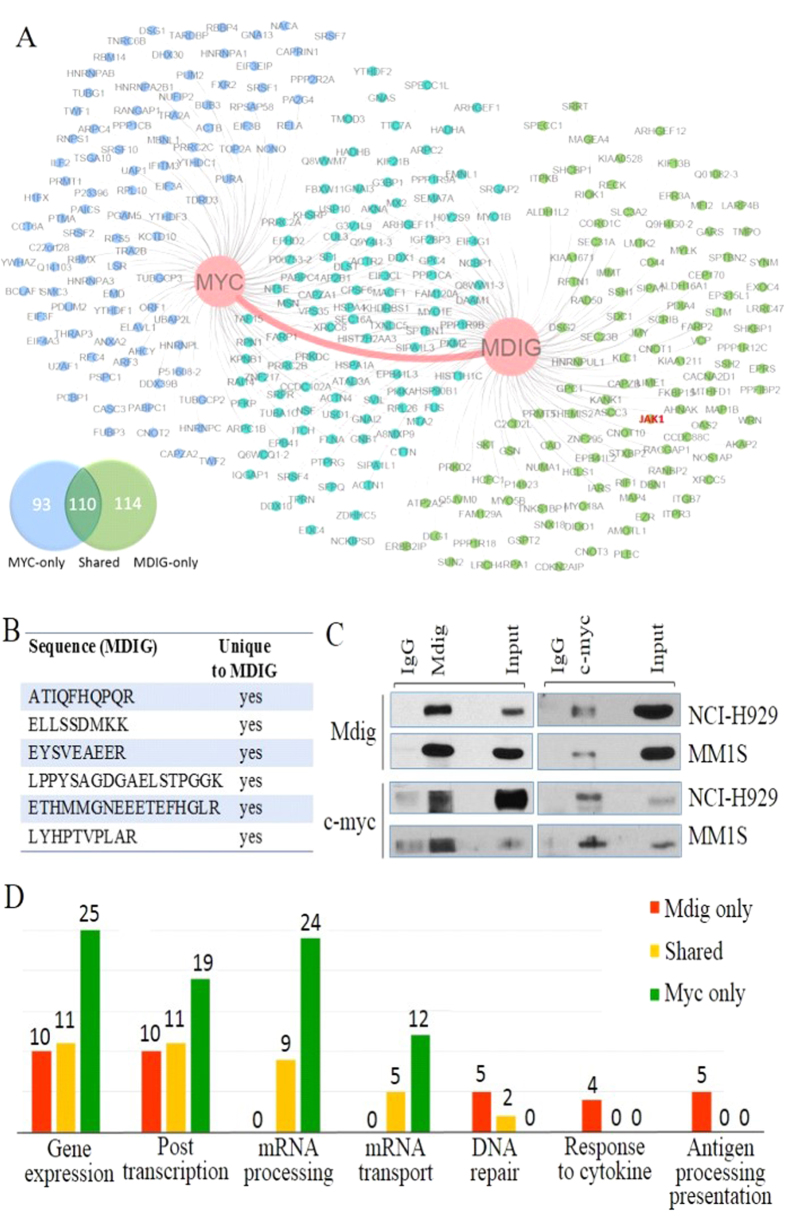
Mdig directly binds to and extensively cooperates with c-myc. (**A**) Proteomic identification of the C-myc-mdig-centered protein interaction network following c-myc and mdig pull-downs. All determined proteins, excluding mdig and c-myc themselves, are categorized as Myc-only (blue), Mdig-only (green) and Shared (cyan) groups while total numbers of each group are listed in the Venn diagram; (**B**) A chart summarizes all the unique peptide sequences of mdig detected by mass spectrometry in c-myc pull-downs; (**C**) Co-immunoprecipitation (co-IP) assay shows direct physical binding of c-myc and mdig in NCI-H929 and MM1S cells; (**D**) Summaries of top biological processes represented by the interaction partners of c-myc and mdig. All determined subjects are interrogated by Gene Ontology database and are sorted based on biological processes they participate in.

In order to decipher inter-regulation between mdig and c-myc in MM cells, proteomics study was performed on MM cell line NCI-H929 cells to screen their interaction partners, respectively. A total of 224 and 203 proteins were identified as significant binding partners of mdig and c-myc, respectively. Among them, 110 binding partners are shared by mdig and c-myc (Fig. 4A). Strikingly, physical binding between mdig and c-myc was detected by mass-spectrometry in NCI-H929 cells (Fig. 4B), which was further validated by co-IP assay in both NCI-H929 and MM1S cells (Fig. 4C), implying that mdig might be assembled into functional protein complexes together with c-myc and directly participate in c-myc-induced oncogenesis for the development of MM. Subsequent network analysis highlighted some major cellular events upon which c-myc and mdig are most likely to impose their impact (Fig. 4D). The shared binding partners are mainly clustered in 4 areas: gene expression, post-transcriptional regulation of gene expression, mRNA processing, and mRNA transport. It is not surprising that c-myc-only binding partners are actively involved in all 4 biological processes and mdig-only binding partners involved in former 2 processes considering the well-established role of c-myc as an essential transcription factor^10^,^37^,^40^ and mdig as an important epigenetic regulator^13^,^14^. Collectively, these data provide a strong rationale that mdig is a core direct interaction partner of c-myc and is most likely to collaborate in gene expression-related functions in MM cells. Notably, mdig-only binding partners are also enriched in proteins important for cellular responses to cytokine and antigen processing and presentation, which is in agreement with our previous findings suggesting that mdig contributes to the function of the T helper 17 (Th17) cells^17^,^41^. Most recently, we discovered that mdig interacts with DNA double strand break repair proteins in the non-homologous end-joining (NHEJ) pathway in human bronchial epithelial cells and lung cancer cells^42^. In MM cells, we also identified at least 7 DNA repair proteins that interact with mdig, including XRCC5, XRCC6, Rad50, etc. (Fig. 4D), indicating that mdig may also be involved in handling cellular stress caused by ongoing DNA damage, a common feature in human MM^36^,^43^.

### Mdig binds JAK1 in MM cells

**Figure 5:**
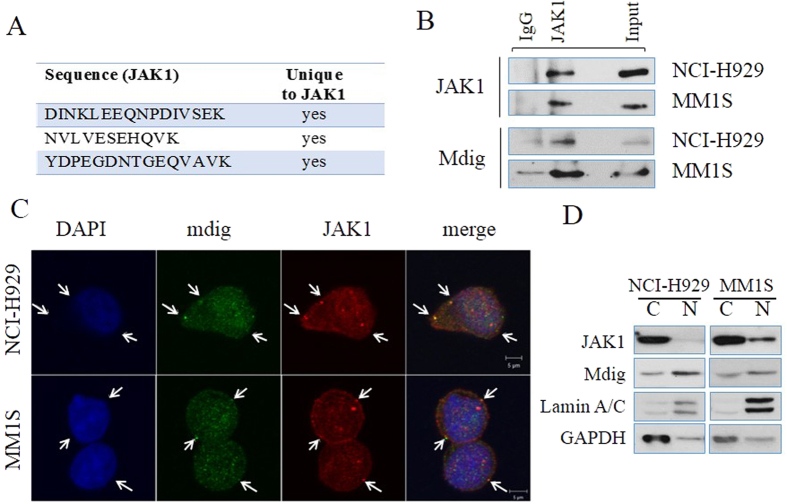
Direct interaction between mdig and JAK1. (**A**) Proteomic identification of the unique peptide sequences of JAK1 detected by mass spectrometry in mdig pull-downs; (**B**) Co-IP assay demonstrates the physical binding between mdig and JAK1 in total cell lysates; (**C**) Confocal microscopy shows co-localization of mdig and JAK1 in NCI-H929 and MM1S cells. Primary antibodies: JAK1 (rabbit anti-human) and mdig (mouse anti-human). Secondary antibodies: Red (goat anti-rabbit) and Green (goat anti-mouse). Sites of co-localization are indicated by arrows; (**D**) Immunoblotting of mdig and JAK1 in nuclear extracts (N) and cytosolic lysates (**C**) in 2 MM cell lines. The volume ratio of final nuclear extracts over cytosolic lysates is 1:4. In this test, cytosolic protein (30 μg) and nuclear protein at identical volume ratio were used to reflect the distribution of target proteins in indicated cellular compartments. Lamin A/C and GAPDH are used as markers for nucleus and cytosol, respectively.

Among the most important signaling pathways, IL-6/JAK/STAT3 signaling has been viewed as an indispensable signal for the malignant transformation of plasma B cells and proliferation of the MM cells^44^. Through cooperation with c-myc, this signaling pathway drives formation of highly malignant MM in mouse model^45^. It is unclear how this cooperation is established between the oncogenic signal and cytokine signal. It is noteworthy that proteomic study identifies JAK1, a key regulator mediating cytokine-induced signaling, as a significant interaction partner of mdig (Fig. 5A). Following this clue, co-IP assay was performed using total cell lysates of both NCI-H929 and MM1S cells and confirmed such a physical interaction (Fig. 5B). The interaction of mdig and JAK1 was additionally verified by immunofluorescent staining and confocal microscopy. Multiple co-localization sites of mdig and JAK1 were observed in the extra-nuclear area in both NCI-H929 cells and MM1S cells (Fig. 5C). Mdig has long been recognized as a nuclear protein^15^,^16^, whereas JAK1 is believed to be a cytosolic protein in the proximity of cytokine receptors. It is interesting to know how a nuclear protein can interact with a cytosolic protein. To answer this question, different cellular compartments were separated through fractionation. Surprisingly, in both MM cell lines, a significant portion of mdig was found in cytosol though the majority of mdig located in nucleus (Fig. 5D). Thus, cytosolic localization of mdig may be accounted for the proximity and physical interaction between mdig and JAK1. This is also the first observation of mdig in cytosol of human cell lines without additional manipulation, although we had also noted cytosolic localization of mdig in mdig-overexpressed or arsenic-treated A549 cells^16^.

### Mdig stabilizes JAK1

**Figure 6:**
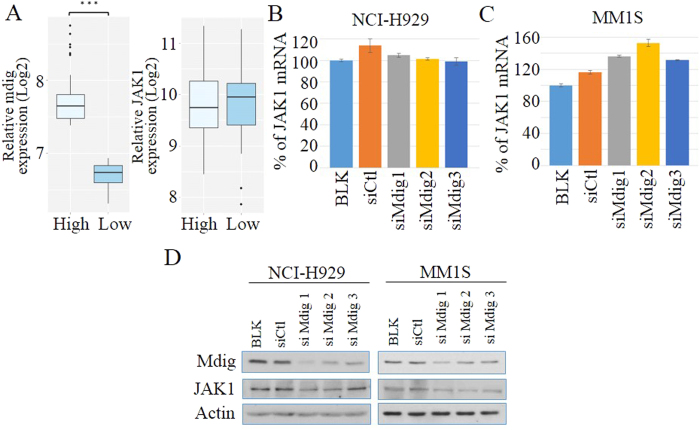
Mdig stabilizes JAK1. (**A**) Correlation analysis of mdig and JAK1 mRNA expressions in MM patients. Methods and parameters used are the same as described in Fig. 3 (***p < 0.001); (**B**,**C**) qRT-PCR shows relative expression levels of JAK1 in NCI-H929 (**B**) and MM1S (**C**) cells treated with control siRNA and 3 different siRNAs against mdig. The values are normalized to blank group (BLK) and displayed as mean ± SD (n = 3, *p < 0.05). (**D**) Immunoblotting analysis of JAK1 expression in 2 MM cell lines treated with control and 3 different mdig siRNAs.

To investigate the biological function of mdig-JAK1 interaction, we further studied the role of mdig on the gene expression and protein stability of the JAK1 protein in MM cells. The co-amplification analysis on MM patients exhibits no significant difference of JAK1 mRNA level between “mdig high” and “mdig low” groups (Fig. 6A). In NCI-H929 cells, genetic silencing of mdig does not affect mRNA level of JAK1 (Fig. 6B), while in MM1S cells, mdig knock-down groups displayed slightly higher JAK1 mRNA expression than the control group (Fig. 6C). However, on the protein level, silencing mdig resulted in a considerable decrease of total JAK1 protein (Fig. 6D). These data suggest that mdig affects the JAK1 protein level through some posttranslational mechanisms. Given the potential activity of mdig on lysine demethylation^13^,^14^, we hypothesize that mdig may regulate JAK1’s stability by removing the methyl groups from its lysine residue(s). Because there is no report of JAK1 methylation so far and the unavailability of antibodies targeting methylated JAK1, we first immunoprecipitated and collected JAK1 protein from the control and mdig-silenced MM cells and then probed the samples with an antibody that selectively recognizes methylated lysine. We showed a marginal lysine methylation on JAK1 in both NCI-H929 and MM1S cells in which mdig was silenced by siRNA (data not shown). However, this set of data remains to be further confirmed.

### Mdig and c-myc are required for the hyperactivation of the IL-6 signaling

**Figure 7:**
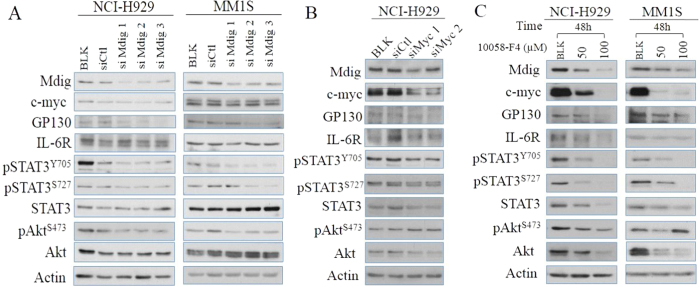
Mdig and c-myc modulates IL-6 signaling. (**A**) Immunoblotting analysis of expression and activity of major regulators involved in IL-6 signaling pathway in human NCI-H929 and MM1S cells treated with control and 3 different mdig siRNAs; (**B**) Immunoblotting analysis of expression and activity of major regulators involved in IL-6 signaling pathway in human NCI-H929 cells in which c-myc was silenced by two different siRNAs. (**C**) Immunoblotting analysis of expression and activity of major regulators involved in IL-6 signaling pathway in human NCI-H929 cells and MM1S cells treated with c-myc inhibitor, 10058-F4, for 48 h.

Synergetic collaborations between c-myc and IL-6 pathways have been well-documented in MM^12^,^37^,^46^. Prompted by the implications from proteomics studies above, we next interrogated the possibility of mdig in mediating the oncogenic crosstalk between c-myc and IL-6 signaling. Consistent with a previous report^47^, our biochemical analysis demonstrated that genetic silencing of mdig results in decreased protein levels of GP130, but not IL-6R in both NCI-H929 and MM1S cell lines (Fig. 7A). Moreover, mdig silencing further leads to attenuated phosphorylation of the major downstream effectors on IL-6 signaling pathway, including STAT3 on both Tyrosine 705 and Serine 727 sites, and Akt on Serine 473 site, but not their total protein levels (Fig. 7A). On the other hand, silencing c-myc or treatment of the cells with a c-myc inhibitor, 10058-F4, leads to a notable decrease of the mdig protein and all regulators on IL-6 pathway mentioned above, except pAkt (Fig. 7B,C), indicating that c-myc is an essential transcription factor in MM cells while mdig specifically cooperates with c-myc in promoting overexpression of GP130 and, consequently, causes amplification of the IL-6 signaling for cell survival and growth.

## Discussion

Considerable progresses in understanding the molecular pathogenesis of MM have been achieved in the past years. However, many important questions remain to be answered, such as the risk factors for MM and the extensive crosstalk between various oncogenic mechanisms in MM. Bone marrow is a complex and dynamic microenvironment with stromal cells, osteoclasts, T lymphocytes, cytokines and growth factors, which are critical for disease evolution of MM. In such a profoundly-intertwined regulatory network of malignancy, oncogene c-myc and cytokine IL-6 have long been viewed as major driving forces for the pathogenesis of MM^36^,^48^,^49^. In the present study, we provide the first evidence showing that WTC dust is potent in inducing mdig in normal B cells and MM cells, and mdig is a key mediator in synergizing c-myc and IL-6 signaling through direct interaction with c-myc and JAK1. By both upregulating and sustaining key regulators in IL-6 pathway, mdig enables MM cells to take advantage of this critical intracellular pathway to achieve abnormal cell proliferation and apoptosis escape. These results explain, at least in part, the mechanisms underlying the observed synergetic collaboration between IL-6 pathway and c-myc in promoting oncogenesis of the plasma cells.

A study by Moline *et al*.^4^ suggested an increased incidence rate and early onset of MM among the first responders who exposed to the WTC dust. A follow-up study by Li *et al*.^5^ on 55,778 people, including rescue workers, recovery workers, and those who lived or worked near the WTC, also found a higher rate of MM, in addition to thyroid and prostate cancers. Both of these studies noted that there were no previous reports indicating association of special occupation, such as those firefighters and police officers, with MM. The WTC dust released from the collapse of the twin towers after 9/11 attack is a mixture of mineral particles, fibers, metals, and chemicals, many of which are established human carcinogens^30^. Since mdig was originally identified as a mineral dust-induced gene from coal workers who exposed to mining and coal dust in a daily basis^21^, we sought to determine whether induction of mdig can be indicative for the association of MM and WTC dust. Indeed, we found that WTC dust is highly capable of inducing mdig expression in either bronchial epithelial cells, normal B cells, or the MM cells. Although the results reported here can be viewed as circumstantial, they may be considered as “proof of principle” to address the carcinogenic potential of environmental factors on the development of MM.

The findings that mdig is strongly associated with the disease progression of MM patients suggest that mdig can be potentially used as a prognostic marker to guide clinical management of the MM patients. A similar role of mdig had been reported in human gastric carcinoma^50^. Our analysis shows that mdig mRNA significantly increases as disease progresses. Notably, the increases of mdig expressions in MM verse MGUS and MM versus SMM are both statistically significant. In addition, high level of mdig is also significantly associated with poor overall survival of the MM patients. In the cellular models, we have demonstrated that genetic silencing of mdig in MM cells leads to constitutive suppression of GP130 (IL-6ST) and pro-survival regulators, STAT3 and Akt, suggesting that mdig inhibition could be a possible strategy to suppress tumor growth in IL-6-dependent MM subtypes or sensitize them to IL-6-targeted agents.

The mdig protein contains a conserved JmjC domain without classic chromatin- or DNA-binding domains^14^,^51^. In accordance with a recent report^42^, our proteomic analysis has unraveled direct interactions of mdig with a number of chromatin-binding proteins and DNA repair proteins. Given that our current findings have clearly demonstrated a regulatory circuit among c-myc, mdig and IL-6 signaling, it is plausible to speculate that mdig may be assembled into protein complexes with chromatin- or DNA-binding protein(s), like c-myc, and be recruited to MM-specific signature genes, including GP130, and exerts its regulatory functions on gene expression. On the other hand, recent studies have discovered that transcription-related regulators can translocate to different cellular compartments and carry out non-canonical functions. For example, Enhancer of Zeste Homolog 2 (EZH2), a well-documented epigenetic silencer for gene transcription, has been shown to directly interact with and methylate STAT3^52^. Similarly, in the present report, we have observed that mdig binds to JAK1 in cytosol, which may stabilizes JAK1 protein.

In summary, we provided the first evidence showing that WTC dust is highly capable of inducing mdig expression in human bronchial epithelial cells, normal human B cells and human MM cell lines. The levels of mdig mRNA and protein are associated with the disease progression and prognosis of the MM patients. Proteomic analyses suggested a direct interaction of mdig with c-myc and JAK1 in MM cell lines, which was responsible for the hyperactivation of the IL-6-JAK-STAT3 signaling important for the pathogenesis of MM. Additional studies are much needed to determine whether the level of mdig expression can be used to guide the clinical managements of the MM.

## Materials and Methods

### Cells and reagents

Human MM cell lines, NCI-H929 and MM1S, bronchial epithelial cell line BEAS-2B, and normal B cell line C5B7 were purchased from American Type Culture Collection (ATCC, Manassas, VA, USA) and maintained in ATCC-recommended culture conditions. Inhibitor of c-myc (10058-F4) was purchased from Sigma-Aldrich Co. (St. Louis, MO, USA). WTC dust was provided by Dr. Kenneth Reuhl at the Environmental and Occupational Health Sciences Institute of the Rutgers University.

### siRNA transfection

Transfections were performed using Lipofectamine RNAiMAX (Invitrogen) according to manufacturer’s protocol. Fifty nM of siRNAs were used for transfection followed by 48-hour incubation. Control siRNA, Mdig siRNAs and c-myc siRNAs were purchased from Qiagen (Valencia, CA, USA).

### Immunohistochemistry (IHC)

Tissue microarray slides, T293 and BM483b, containing multiple myeloma samples and non-cancerous bone marrow tissue were purchased from US Biomax, Inc (Rockville, MD). IHC staining was performed as previously described^23^. Briefly, the slides were stained overnight at 4 °C with mouse anti-human mdig antibody (Invitrogen) at 1:50 dilution followed by biotinylated goat anti-mouse secondary antibody (Dako Denmark A/S, Glostrup, Denmark) at 1:200 dilution for 2 hours at room temperature. The slides were then incubated with ABC reagent and DAB (Vector Laboratories, Inc. Burlingame, CA), counter stained with hematoxylin and mounted with entellan. All images were captured using a Nikon Eclipse Ti-S Inverted microscope (Mager Scientific, Dexter, MI). Cut-offs between positive and negative cells was determined according to previously characterized mdig-expressing breast cancer samples^23^. Four random images were taken for each sample and both positive and negative cells were counted using ImageJ 1.48 v ( http://imagej.nih.gov/ij/). Mdig expression status of all samples was classified into four grades based on the percentage of positively-stained cells. Strongly positive: over 50%; moderately positive: between 50% and 25%; weakly positive: between 25% and 5%; negative: less than 5%.

### Immunoblotting and Immunoprecipitation (IP)

Immunoblotting and IP analysis were performed as previously reported^42^. NE-PER Nuclear Cytoplasmic Extraction KIT (Thermo Scientific Pierce, Rockford, IL, USA) was used to isolate nuclear proteins. Densitometric analysis of CHX-treated samples was completed using ImageJ 1.48 v ( http://imagej.nih.gov/ij/). When detecting c-myc bands in IP samples, HRP-conjugated protein A (EMD Millipore, Temecula, CA, USA) was used to minimize the background noise caused by IgG heavy chain. Primary antibodies against phospho-Akt (Ser473), total Akt, phospho-STAT3 (Ser727), phospho-STAT3 (Tyr705), total STAT3, phospho-JAK1 (Tyr1022), total JAK1, GAPDH, actin and all secondary antibodies were purchased from Cell Signaling Technology (Danvers, MA, USA). Antibodies against GP130, IL-6R and methylated-lysine were purchased from Abcam (Cambridge, MA). Antibodies against c-myc and lamin A/C were purchased from Santa Cruz Biotechnology (Dallas, Texas, USA). Mdig (mouse) antibody was ordered from Invitrogen. Antibodies used for IP include Mdig (rabbit) and c-myc (mouse) from Abcam (Cambridge, MA, USA), c-myc (rabbit) from Cell Signaling Technology (Danvers, MA, USA), JAK1 (rabbit) from Santa Cruz Biotechnology (Dallas, Texas, USA). All presented data are representatives of at least 3 independent experiments.

### Confocal immunofluorescence (IF) analysis

For IF staining, 10^6^ cells were centrifuged, fixed by 4% formaldehyde for 15 min, permeabilized by 0.3% Triton X-100 and blocked in PBS containing 5% normal goat serum and 0.1% Tween 20 for 1 hour at room temperature. Then they were incubated with primary antibodies, JAK1 (rabbit, Santa Cruz Biotechnology) and Mdig (mouse, Invitrogen) overnight at 4 °C and with Invitrogen secondary antibodies, Alexa Fluor 488-linked antibody (goat anti-mouse) and Alexa Fluor 594-linked antibody (goat anti-rabbit) for 1 h at room temperature in dark. All antibodies were used at 1:100 dilution. Prolong Gold antifade reagent with DAPI (Invitrogen) was used to preserve the samples. Co-localization of JAK1 and Mdig was detected by Zeiss LSM 780 confocal microscope (Carl Zeiss Microscopy, Jena, Germany). Pinhole size of 60 μm was used while thresholds for laser power, master gain and digital gain were determined by non-specific binding controls. DAPI, Alexa Fluor 488 and Alexa Fluor 594 were excited at 405 nm, 488 nm and 595 nm and corresponding fluorescence emissions were detected at 495 nm, 563 nm and 640 nm via 3 independent channels. All photos were processed using ZEN 2012 SP1 64 bit software (Carl Zeiss Microscopy, Jena, Germany).

### PCR

Total RNAs were extracted using TRIzol Reagent (Life Technologies, Grand Island, NY, USA) and their integrity was assessed by 18S and 28S ribosomal RNAs. For reverse transcription PCR, AccessQuick RT-PCR system from Promega (Madison, WI) was used. The primers for mdig are: 5′-TCA TGT CGG GCC TAA GAG AC-3′ and 5′-GGC ATT TGA TTC TGC AAA GG-3′, which amplifies a 1,510 bp DNA fragment covering the whole coding region of the mdig gene. Primers for GAPDH are: 5′-CTG AAC GGG AAG CTC ACT GGC ATG GCC TTC-3′ and 5′-CAT GAG GTC CAC CAC CCT GTT GCT GTA GCC-3′. For real-time PCR, one μg total RNAs were reverse-transcribed using High-Capacity cDNA Reverse Transcription Kit (Applied Biosystems, Waltham, MA, USA) and 1:20 diluted. Jak1 and ACTB Taqman Gene Expression Assays (Best Coverage) were purchased from Applied Biosystems (Waltham, MA, USA). Samples were run in triplicates, quantified by ΔΔCt method with actin as reference gene and normalized to “Blank” group. Final results were shown as mean ± SD.

### Mass spectrometry and proteomics analysis

Proteomics profiling of binding partners were performed as previously reported^42^. Briefly, samples were subject to co-IP, 1D-SDS-PAGE separation, in-gel digestion, peptide purification and HPLC-ESI-MS/MS analysis. Protein identity was determined by MaxQuant software.

### Biostatistics analysis

Protein interaction network analysis was completed using Gene Ontology database and visualized by Cytoscape 3.2. Binding proteins were first sorted according to their biological processes and further refined manually by merging repeating and redundant categories. Gene expression data were accessed through Multiple Myeloma Genomics Portal ( https://www.broadinstitute.org/mmgp/home) for GSE6477 and through GEO for GSE39754 and GSE2658 before being processed and visualized using R project with ggplot2 package. Survival analysis in Fig. 3E was performed using Kaplan-Meier method and differences between 2 cohorts were determined using log-rank test. In Fig. 3C,D, differences of mRNA levels between patient cohorts were calculated using one-way ANOVA and p-values were adjusted by Holm method. All other mRNA expression comparisons were performed using two-tailed t-test. Considering that expression levels of related genes are not always strictly linear to each other, we conducted “Force Rank” co-amplification analysis as previously described^53^. A p-value less than 0.05 is considered statistically significant.

## Additional Information

**How to cite this article**: Wu, K. *et al*. Proteomic Characterization of the World Trade Center dust-activated mdig and c-myc signaling circuit linked to multiple myeloma. *Sci. Rep.* **6**, 36305; doi: 10.1038/srep36305 (2016).

**Publisher’s note:** Springer Nature remains neutral with regard to jurisdictional claims in published maps and institutional affiliations.
